# Supplementary material for: Cripto shapes macrophage plasticity and restricts EndMT in injured and diseased skeletal muscle
Source: EMBO Rep. 2020 Feb 27;21(4):e49075. doi: 10.15252/embr.201949075 (PMC7132341; doi:10.15252/embr.201949075)
Supplement: Supplementary file 1 — Appendix [file EMBR-21-e49075-s001.pdf]

Appendix

Cripto shapes macrophage plasticity and restricts EndMT in injured and diseased skeletal muscle

Francescopaolo Iavarone[1], Ombretta Guardiola[1], Alessandra Scagliola[2], Gennaro Andolfi[1], Federica Esposito[1], Antonio Serrano[3], Eusebio Perdiguero[3], Silvia Brunelli[2], Pura Muñoz-Cánoves[3,4,5] and Gabriella Minchiotti[1]*

1. *Stem Cell Fate Laboratory, Institute of Genetics and Biophysics "A. Buzzati-Traverso", CNR, 80131, Naples, Italy.*

2. School of Medicine and Surgery, University of Milano-Bicocca.

3. Cell Biology Group, Department of Experimental and Health Sciences, Pompeu Fabra University (UPF), CIBER on Neurodegenerative Diseases (CIBERNED), Barcelona, Spain.

4. Institució Catalana de Recerca i Estudis Avançats (ICREA), Barcelona, Spain.

5. Centro Nacional de Investigaciones Cardiovasculares (CNIC), Madrid, Spain.

*Correspondence to: Gabriella Minchiotti, gabriella.minchiotti@igb.cnr.it

Table of contents:

Appendix Figure S1 ……………………………………………………………………..……..2
Appendix Figure S2 ……………………………………………………………..………..3
Appendix Figure S3 ……………………………………………………………..………..4
Appendix Figure S4 ………..……………………………………………………………..5
Appendix Table S1 ……..…… ……………………………………………………..…..6
Appendix Table S2 ……..………………..………………………………………..………7
Appendix Table S3 ………………………..……………………………………………..…8

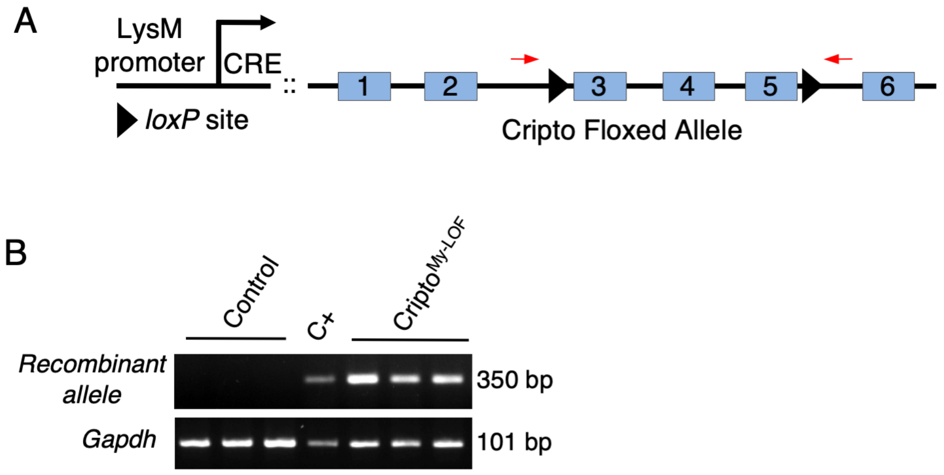

Appendix Figure S1.

A. Schematic representation of *Tg:LysMCre::Cripto*$^{fl/fl}$ (Cripto$^{My\text{-}LOF}$) transgenic mice. Cre is under the control of the Lysozyme M (LysM) promoter and induces the tissue-specific excision of exon 3-5 in *Cripto* floxed allele. Red arrows indicate forward and reverse primers for PCR genotyping.

B. PCR genotyping on DNA extracted from CD11b+ MPs FACS-isolated from Cripto$^{My\text{-}LOF}$ and Control at day 2 after injury. *Gapdh* was used as control.

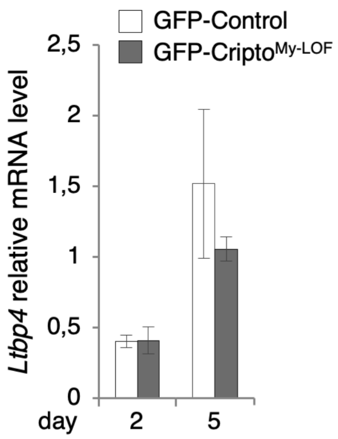

Appendix Figure S2.

QRT-PCR analysis of *Ltbp4* in FACS-isolated GFP+ MPs from GFP-Cripto$^{My\text{-}LOF}$ and GFP-Control muscles at day 2 and 5 after injury. Data are mean±SEM of relative mRNA level normalized with Gapdh (n=4 biological replicates; P=ns; Student t-test).

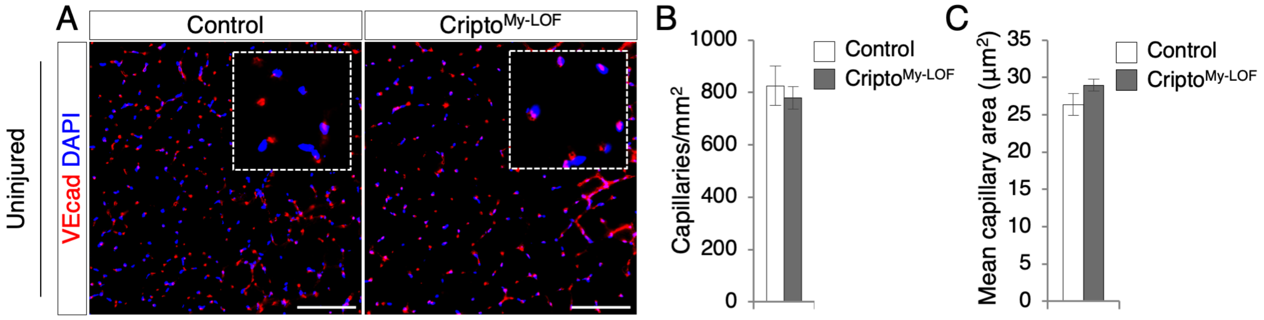

Appendix Figure S3.

A. Representative pictures of VEcad (red) immunostaining of Cripto$^{My\text{-}LOF}$ and control TA sections in resting conditions. Nuclei were counterstained with DAPI (blue). Scale bar is 100 µm. Magnification of the boxes is 3.5 x.

B-C. Quantification of VEcad+ capillary number (B) per area (mm^2) and average of capillary cross-sectional area (µm2; C). Data are mean±SEM (n=5 biological replicates; P=ns; Student *t*-test).

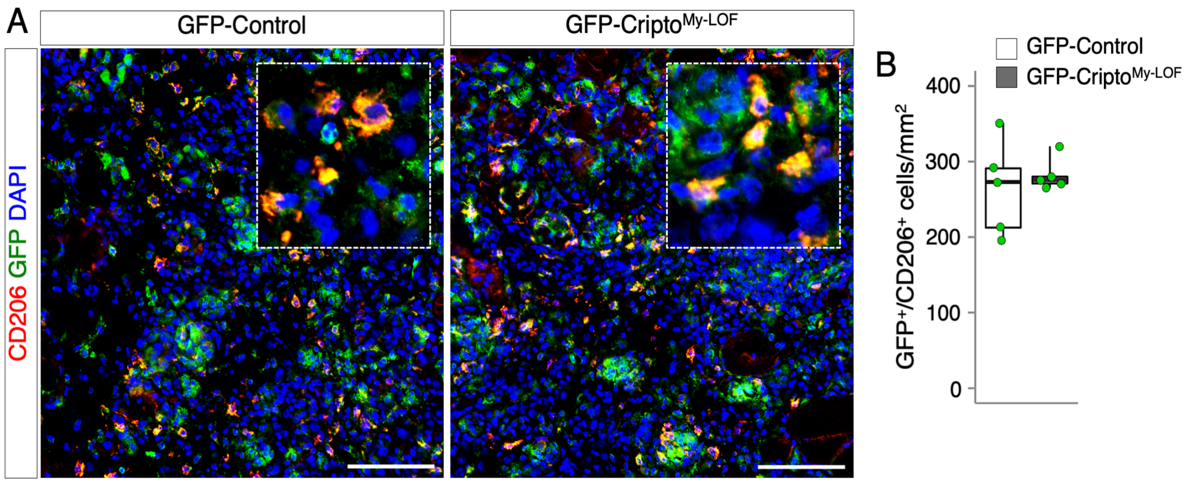

Appendix Figure S4.

A. Representative pictures of double immunostaining with CD206 (red) and GFP (green) on GFP-Control and GFP-Cripto^{My-LOF} TA sections at day 3 after injury. Nuclei were counterstained with DAPI (blue). Scale bar is 100 µm. Magnification of the boxes is 3.5 x.

B. Quantification of GFP/CD206 double positive cells per area (mm^2). Data are expressed as box plots (n=5 biological replicates; P=ns; Student *t*-test).

Appendix Table S1. The list of Primers for genotyping

Allele	Primer 1 (5'-3')	Primer 2 (5'-3')	PCR Amplification (bp)
Cripto	TCTGCACTGGGGCTAAACCTTATG	GCCAAGAGCCATGACAGAGATGG	380
Cripto$^{fl/fl}$	TCTGCACTGGGGCTAAACCTTATG	GCCAAGAGCCATGACAGAGATGG	*580*
Cripto^{del}	TCTGCACTGGGGCTAAACCTTATG	CATCTGGGACATGCCCACTA	350
LysM	CTTGGGCTGCCAGAATTTCTC	CCCAGAAATGCCAGATTACG	350
LysMCre	CTTGGGCTGCCAGAATTTCTC	TTACAGTCGGCCAGGCTGAC	700
R26 WT	AAAGTCGCTCTGAGTTGTTAT	GGAGCGGGAGAAATGGATATG	*650*
R26^{mTmG}	AAAGTCGCTCTGAGTTGTTAT	GTCGTTGGGCGGTCAG	250
Cripto (reference PCR)	TACACTGCTTCATCCCC	TAGCAGCCCTAAGTGTC	156
Cripto (undeleted allele)	CTCCTGATGCCACTTCATGC	GCCCATAAGCAACGGTTCAT	171

Appendix Table S2. The list of Primers used for qRT-PCR expression analysis

Gene	Forward (5'-3')	Reverse (5'-3')
Arg1	AGCTCCAAGCCAAAGTCCTTAGA	CCTCCTCGAGGCTGTCCTT
Cripto	GCATTTGGGACCAGAAAGAA	GCGTCCATAGAAGGAAGGAG
Fizz1	TCCAGCTAACTATCCCTCCACTGT	GGCCCATCTGTTCATAGTCTTGA
Gapdh	TCTTCTGGGTGGCAGTGATG	TGCACCACCAACTGCTTAGC
Il10	ATTTGAATTCCCTGGGTGAGAAG	CACAGGGGAGAAATCGATGACA
Il4rα	CCTCTGTGGGCTGTCTGATTTT	GGGCTCACCCAGGACCTT
Ltbp4	AATTTCCCCTGCCCAGCATC	ACAAACCTGGGGATTTCGCT
Mcp1	CCCAATGAGTAGGCTGGAGA	GCTAAGACCTTAGGGCAGA
Nos2	AGCCAAGCCCTCACCTACTT	TCTCTGCCTATCCGTCTCGT
Tgfβ	CCCCACTGATACGCCTGAGT	AGCCCTGTATTCCGTCTCCTT
Tnfα	TCCCAGGTTCTCTTCAAGGGA	GGTGAGGAGCACGTAGTCGG

Appendix Table S3. The list of Antibodies and their applications

Antibody (Clone)	Species	Dilution	Company (Catalog #)	Application
CD11b-PerCP (M1/70)	Rat	1:10	R&D System (FAB1124C)	FACS
F4/80-APC (T45-2342)	Rat	1:100	BD (565853)	FACS
Ly6C-PECy7 (AL-21)	Rat	1:200	BD (560593)	FACS
Cripto-488 (237603)	Rat	1:10	R&D System (FAB1538G)	FACS
F4/80 (Cl:A3-1)	Rat	1:25	Bio-Rad (MCA497B)	IF
CD86 (GL1)	Rat	1:50	BD (550542)	IF
CD206 (MR5D3)	Rat	1:200	Bio-Rad (MCA2235)	IF
GFP	Chicken	1:1000	Abcam (ab13970)	IF
Phospho-Smad3 (Ser423/425) (C25A9)	Rabbit	1:100	Cell Signaling (9520)	IF
CD144 (11D4.1)	Rat	1:250	BD (550548)	IF
KLF4	Goat	1:250	R&D System (AF3158)	IF
TCF4 (C48H11)	Rabbit	1:100	Cell Signaling (2569)	IF
CD31 (MEC 13.3)	Rat	1:250	BD (553370)	IF
PDGF Receptor α (D13C6)	Rabbit	1:100	Cell Signaling (5241)	IF
Laminin	Rabbit	1:400	Sigma-Aldrich (L9393)	IF
Embryonic myosin heavy chain	Mouse	1:50	DSHB (F1.652)	IF
Pax7	Mouse	1:20	DSHB	IF
Alexa Fluor 488 Donkey anti-Rat IgG	Donkey	1:500	Invitrogen (A-21208)	IF
Cy3 Donkey Anti-Rat IgG	Donkey	1:500	Jackson IR (712-165-150)	IF
Alexa Fluor 647 Goat anti-Rat IgG	Goat	1:500	Invitrogen (A-21247)	IF
Alexa Fluor 488 Donkey anti-Chicken IgY	Donkey	1:500	Jackson IR (703-545-155)	IF
Alexa Fluor 647 Donkey anti-Rabbit IgG	Donkey	1:500	Invitrogen (A31573)	IF
Alexa Fluor 488 Donkey anti-Rabbit IgG	Donkey	1:500	Invitrogen (A21206)	IF
Alexa Fluor 594 Donkey anti-Rabbit IgG	Donkey	1:500	Invitrogen (A21207)	IF
Alexa Fluor 488 Donkey anti-Goat IgG	Donkey	1:500	Invitrogen (A11055)	IF
Alexa Fluor 594 Donkey anti-Mouse IgG	Donkey	1:500	Invitrogen (A21203)	IF
Biotin-SP Goat anti-Mouse IgG	Goat	1:500	Jackson IR (115-065-205)	IF
