# Supplementary material for: Cripto shapes macrophage plasticity and restricts EndMT in injured and diseased skeletal muscle
Source: EMBO Rep. 2020 Feb 27;21(4):e49075. doi: 10.15252/embr.201949075 (PMC7132341; doi:10.15252/embr.201949075)
Supplement: Supplementary file 2 — Expanded View Figures PDF [file EMBR-21-e49075-s002.pdf]

Expanded View Figures

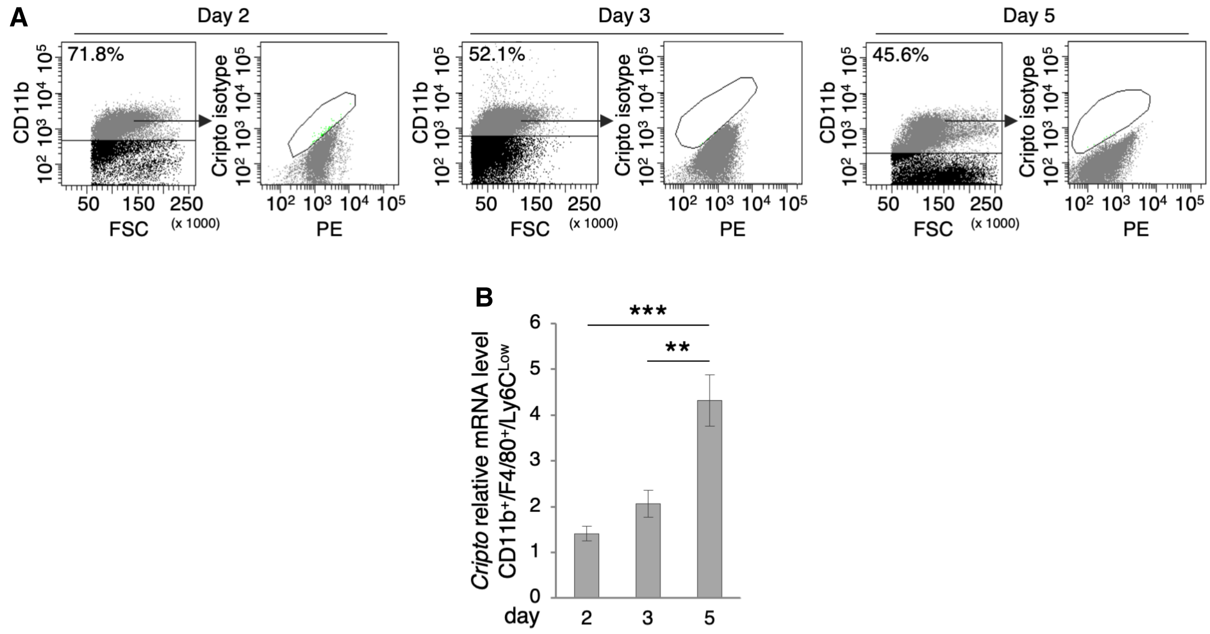

Figure EV1. Cripto isotype control and Cripto expression by qRT–PCR.

A Representative flow cytometry dot plots of Cripto isotype control in $CD11b^+$ cells at days 2, 3, and 5 after injury. PE (Phycoerythrin/free channel).

B Quantitative real-time PCR analysis of Cripto expression profile in $Cd11b^+/Ly6C^{Low}$ MPs at days 2, 3, and 5 after injury. Relative mRNA levels were normalized to *Gapdh*. Data are mean ± SEM ($n = 6$ biological replicates; $^{**}P < 0.01$; $^{***}P < 0.00$, Student's t-test).

© 2020 The Authors

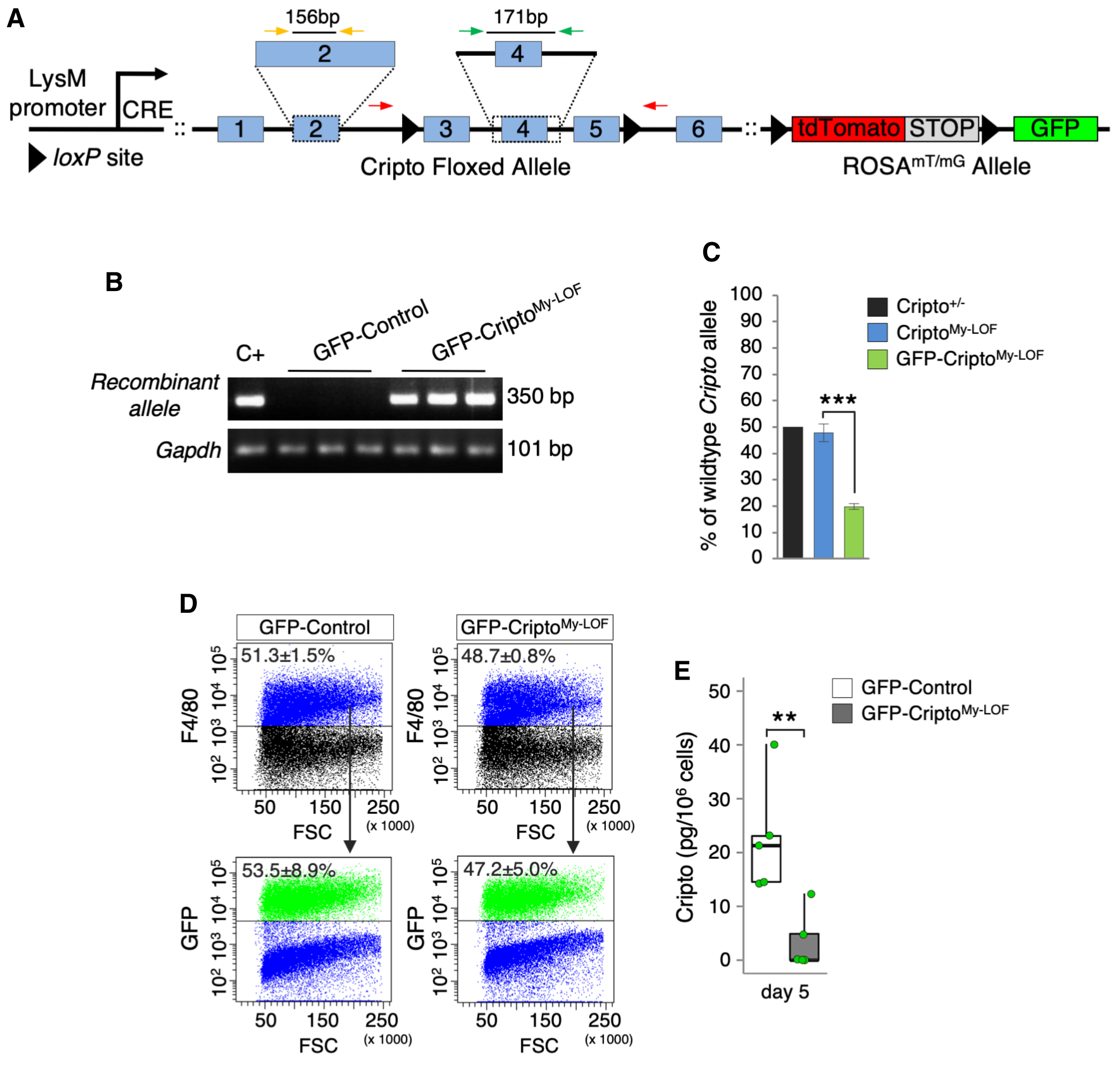

Figure EV2. Efficiency of Cripto deletion.

A Schematic representation of *Tg:LysMCre::R26*^{mTmG}*::Cripto*$^{fl/fl}$ (GFP-Cripto$^{My\text{-}LOF}$) transgenic mice. Red arrows indicate forward and reverse primers used for PCR genotyping. Green arrows indicate forward and reverse primers used to amplify a DNA region encompassing exon 4. Yellow arrows indicate forward and reverse primers used to amplify exon 2, as reference PCR.

B PCR genotyping on DNA extracted from FACS-isolated GFP$^+$ cells from GFP-Cripto$^{My\text{-}LOF}$ and GFP-Control mice at day 2 after injury. *Gapdh* was used as control.

C Genomic quantitative real-time PCR (qRT–PCR) analysis of wild-type *Cripto* allele on FACS-isolated F4/80$^+$ (blue bar) or GFP$^+$ (green bar) MPs from Cripto$^{My\text{-}LOF}$ and GFP-Cripto$^{My\text{-}LOF}$ muscles, respectively, at day 2 after injury. Genomic DNA extracted from Cripto KO heterozygous mice (Cripto$^{+/-}$; black bar) was used as reference of 50% *Cripto* deletion. Data are expressed as percentage of wild-type allele over the reference PCR and are mean ± SEM (n = 6 biological replicates; ***P < 0.000004; Student's t-test).

D Representative flow cytometry dot plots showing the percentage of F4/80$^+$ cell population and GFP$^+$ cells expressing F4/80 in GFP-Control and GFP-Cripto$^{My\text{-}LOF}$ muscles at day 2 after injury. Data are mean ± SEM (n = 4 biological replicates; P = ns; Student's t-test).

E ELISA-based assay of Cripto protein from total protein extracts of FACS-sorted GFP$^+$ MPs from GFP-Control and GFP-Cripto$^{My\text{-}LOF}$ muscles at day 5 after injury. Data are expressed as box plots displaying minimum, first quartile, median, third quartile, and maximum (n = 5 biological replicates; **P < 0.01; Student's t-test).

© 2020 The Authors

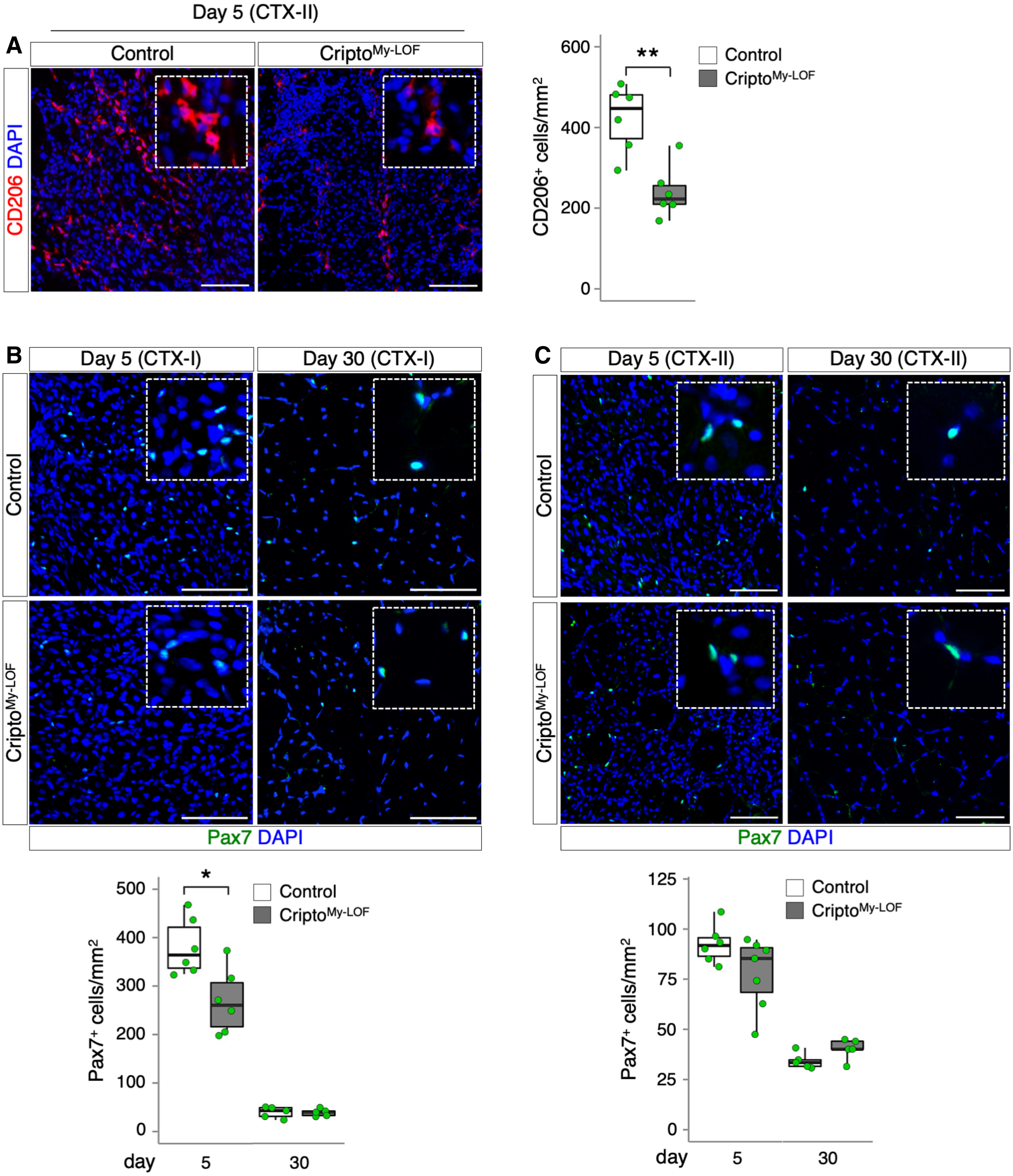

Figure EV3. Effect of Cripto$^{My\text{-}LOF}$ on CD206^{+} MPs and Pax7^{+} satellite cells in acute injury.

A Representative pictures of CD206 (red) immunostaining of Cripto$^{My\text{-}LOF}$ and Control TA sections at day 5 after re-injury (left panel) and quantification of CD206^{+} cells per area (mm^2, right panel). Data are expressed as box plots displaying minimum, first quartile, median, third quartile, and maximum (n = 6 biological replicates; $^{**}P < 0.01$; Student's t-test).

B Representative pictures of Pax7 (green) immunostaining of Cripto$^{My\text{-}LOF}$ and Control TA sections at days 5 (left panel) and 30 (right panel) after injury (CTX-I) and quantification of Pax7^{+} cell number per area (mm^2). Data are expressed as box plots displaying minimum, first quartile, median, third quartile, and maximum ($n \geq 5$ biological replicates; $^{*}P < 0.05$; Student's t-test).

C Representative pictures of Pax7 (green) immunostaining of Cripto$^{My\text{-}LOF}$ and Control TA sections at days 5 (left panel) and 30 (right panel) after re-injury (CTX-II) and quantification of Pax7^{+} cell number per area (mm^2). Data are expressed as box plots displaying minimum, first quartile, median, third quartile, and maximum ($n \geq 5$ biological replicates; P = ns; Student's t-test). Data information: Nuclei were counterstained with DAPI (blue). Scale bar: 100 μm. Magnification of the boxes is 3.5×.

© 2020 The Authors

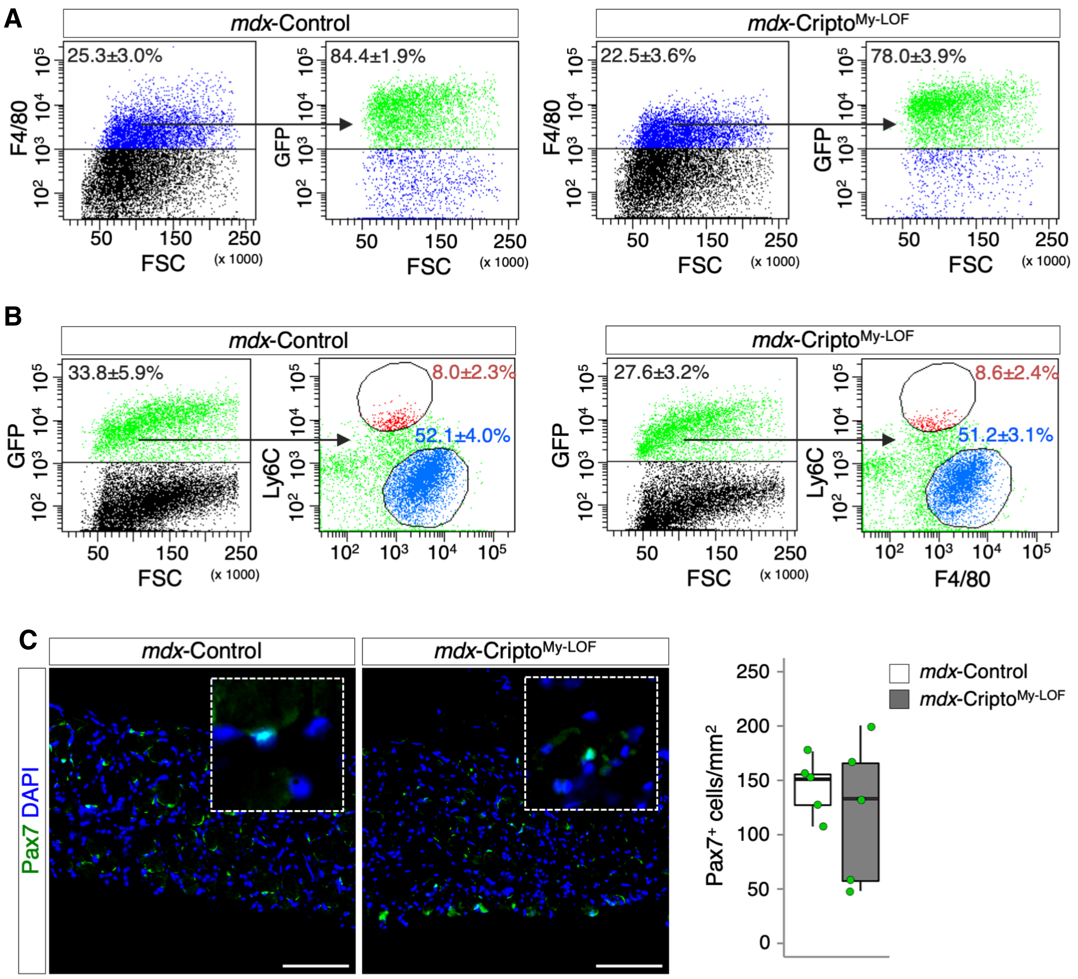

Figure EV4. Cripto$^{My\text{-}LOF}$ does not affect Ly6C$^{high/Low}$ distribution and satellite cells number in mdx mice.

A Representative FACS scatter plots showing the percentage of GFP^{+} gated on the F4/80^{+} cell population in *mdx*-Control and *mdx*-Cripto$^{My\text{-}LOF}$ muscles. Data are mean ± SEM [*n* = 3 biological replicates (*mdx*-Control) and *n* = 5 biological replicates (*mdx*-Cripto$^{My\text{-}LOF}$); *P* = ns; Student's *t*-test].

B Representative flow cytometry dot plots showing GFP^{+} and GFP^{+}/F4/80^{+}/Ly6C$^{Low/High}$ cell populations in *mdx*-Control and *mdx*-Cripto$^{My\text{-}LOF}$ muscles. Data are mean ± SEM [*n* = 3 (*mdx*-Control) and *n* = 5 biological replicates (*mdx*-Cripto$^{My\text{-}LOF}$); *P* = ns; Student's *t*-test].

C Representative pictures of Pax7 (green) immunostaining of *mdx*-Cripto$^{My\text{-}LOF}$ and *mdx*-Control diaphragm sections (left panels) and quantification of Pax7^{+} cell number per area (mm^{2}, right panels). Nuclei were counterstained with DAPI (blue). Scale bar: 100 μm. Magnification of the boxes is 3.5×. Data are expressed as box plots displaying minimum, first quartile, median, third quartile, and maximum (*n* = 5 biological replicates; *P* = ns; Student's *t*-test).

© 2020 The Authors

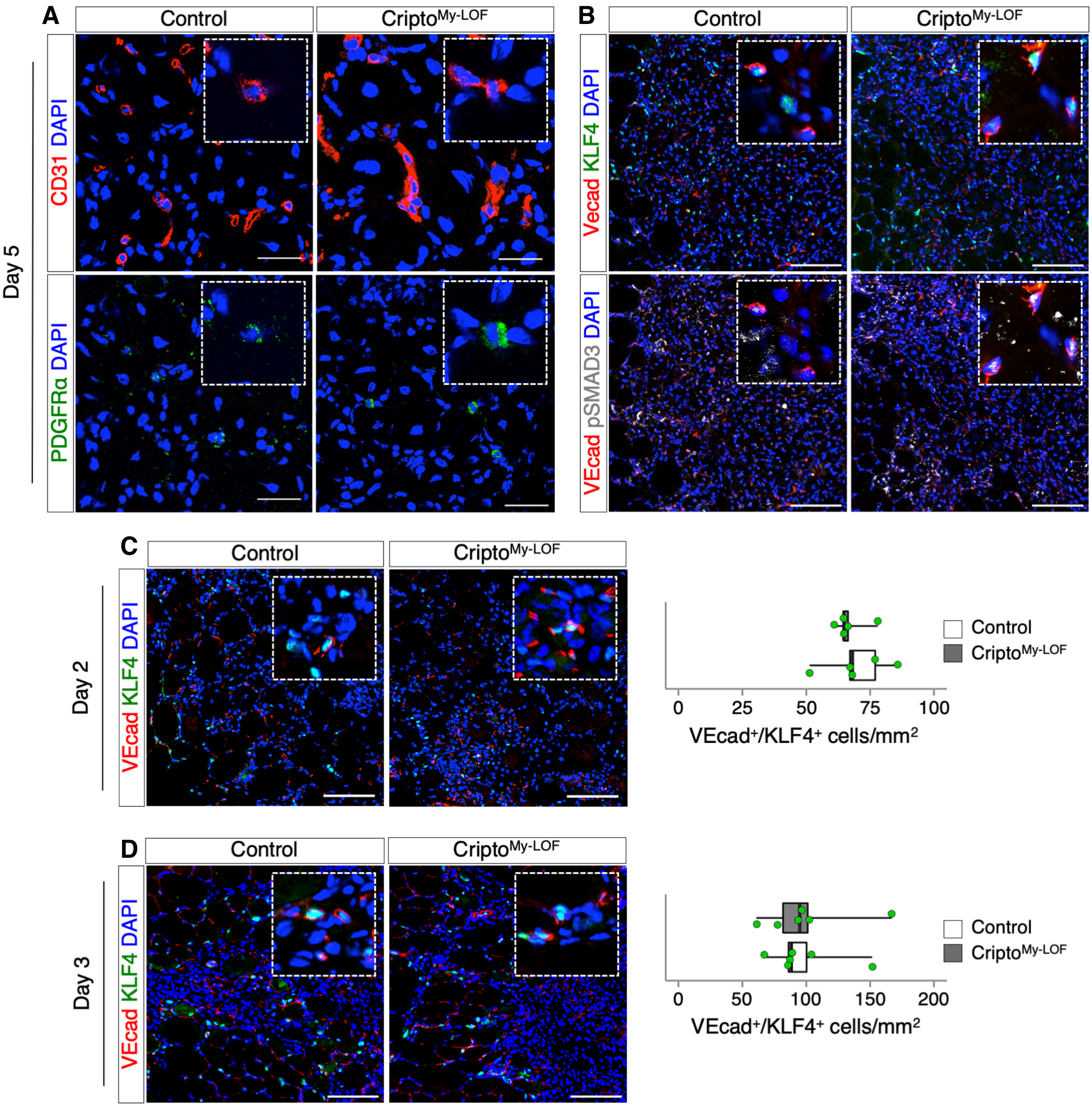

Figure EV5. Cripto^{My-LOF} affects endothelial plasticity in injured skeletal muscle.

A Representative confocal pictures of double immunostaining with CD31 (red) and PDGFRα (green) on Control and Cripto^{My-LOF} TA sections at day 5 after injury. Split green and red channels of Fig 7E are shown.

B Representative pictures of triple immunostaining with VEcad (red), KLF4 (green), and pSMAD3 (white) on Control and Cripto^{My-LOF} TA sections at day 5 after injury. Merged channels of VEcad/KLF4 (top panels) and VEcad/pSMAD3 of Fig 7H are shown.

C, D Representative pictures of double immunostaining with VEcad (red) and KLF4 (green) on Control and Cripto^{My-LOF} TA sections at day 2 (C, left panels) and day 3 (D, left panels) after injury and quantification of VEcad/KLF4 double-positive cells per area (mm^2) at day 2 (C, right panel) and day 3 (D, right panel).

Data information: Nuclei were counterstained with DAPI (blue). Scale bar: 100 µm. Confocal pictures (A) scale bar: 25 µm. Magnification of the boxes is 3.5×. Data are expressed as box plots displaying minimum, first quartile, median, third quartile, and maximum ($n \geq 5$ biological replicates; P = ns; Student's t-test).

 © 2020 The Authors
